# Sleep regularity and body mass index: findings from a prospective study of first-year college students

**DOI:** 10.1093/sleepadvances/zpac004

**Published:** 2022-02-02

**Authors:** Patricia M Wong, David Barker, Brandy M Roane, Eliza Van Reen, Mary A Carskadon

**Affiliations:** 1 Department of Psychiatry and Human Behavior, Alpert Warren Medical School of Brown University, Providence, RI 02903, USA; 2 Sleep for Science Research Laboratory of Brown University, Providence, RI 02903, USA; 3 Department of Pharmacology and Neuroscience, Graduate School of Biomedical Sciences, University of North Texas Health Science Center, Fort Worth, TX 76107, USA; 4 Circadian Positioning Systems, Inc., Providence, RI 02818, USA; 5 E.P. Bradley Hospital, Sleep Research Laboratory, Providence, RI 02906, USA

**Keywords:** sleep regularity, circadian phase, weight gain, obesity risk, young adults

## Abstract

**Study Objectives:**

Using data from a large, prospective study of sleep in first-year college students, we examined whether students’ sleep regularity is associated with body mass index (BMI) and BMI change (∆BMI) during their first college semester. In a subset of participants, we also tested whether dim light melatonin onset (DLMO) phase and DLMO-bedtime phase angle are associated with BMI and ∆BMI.

**Methods:**

Analyses included data from 581 students (mean age = 18.7 ± 0.5 years; 58% female; 48% non-white) who had their height and weight assessed at the start of classes (T1) and end of 9 weeks. Participants completed online daily sleep diaries from which total sleep time (TST) and the sleep regularity index (SRI) were calculated. Among participants who completed a DLMO protocol (*n* = 161), circadian phase was quantified by DLMO and circadian alignment by DLMO-bedtime phase angle. Data were analyzed with linear regressions that controlled for sex and average TST.

**Results:**

Average SRI was 74.1 ± 8.7 (range: 25.7; 91.6). Average BMI at T1 was 22.0 ± 3.5 and participants gained 1.8 ± 2.4 kg (range: −7.2; 11.4); 39% gained 2–5 kg, 8% gained >5 kg. Lower SRI was associated with greater BMI at T1 (*B* = −0.06 [95% CI: −0.09; −0.02], p = 0.001) but not with ∆BMI (p = 0.062). Average TST was not significantly associated with BMI or ∆BMI, nor were circadian phase and alignment in the subsample (p’s > 0.05).

**Conclusions:**

Sleep regularity is an understudied but relevant sleep dimension associated with BMI during young adulthood. Our findings warrant future work to examine longer-term associations between sleep regularity and weight gain.

**Statement of Significance**

The current study shows an association between sleep irregularity and body mass index among young adult college students while quantifying sleep regularity with the sleep regularity index, a metric that considers day-to-day variability in participants’ sleep patterns. Findings suggest that regularity of the sleep-wake cycle is an important but understudied dimension of sleep relevant to obesity risk. Although we did not find a significant association between sleep regularity and change in BMI over time, future studies are needed to consider individual demographic, sleep, and circadian characteristics that may modify effects of sleep regularity, as well as longer-term changes in BMI throughout young adulthood.

## Introduction

Obesity persists as a leading risk factor for medical morbidity and mortality, and recent data indicate that 20% of adolescents and 39.8% of adults in the United States are obese [1]. This high prevalence of obesity is rising in the general population, and particularly among adolescents and young adults [1]. Identifying modifiable behavioral and lifestyle factors that contribute to weight gain earlier in life, particularly during the transitional stage to young adulthood, may inform preventative strategies.

Before the COVID-19 pandemic, an estimated 69% of high school students transitioned immediately to college [2]. Young adulthood for many American college students involves the development of lifestyle habits, including sleep behaviors that may contribute to their long-term obesity risk. As reviewed elsewhere, shorter sleep duration assessed across the lifespan has been linked to greater body mass index (BMI) and weight gain over time [3, 4]. Although a large literature has focused on the role of sleep duration, sleep is multidimensional and good sleep health involves not only adequate sleep duration but also appropriate timing and regularity of sleep schedules [5, 6]. Sleep is regulated in part by the circadian timing system but is also a behavior that individuals can modify. Yet, due to societal obligations such as school or work schedules, individuals may sleep at times that are misaligned to their internal circadian phase [7]. In addition, individuals who frequently shift their sleep times likely experience frequent shifts in the timing of their daily light exposure, which can further contribute to circadian misalignment [8]. Consequences of circadian misalignments include alterations in energy balance, glucose regulation, inflammation, hormonal satiety signaling, and other physiological processes that together can contribute to obesity and its sequalae [9–14]. Understanding how sleep regularity, circadian phase, and circadian alignment relate to obesity risk among college students may elucidate additional dimensions of sleep to target in young adults for weight management.

Although preliminary evidence suggests that sleep timing and regularity of sleep intervals are associated with obesity risk, the assessments of sleep regularity used in previous studies are somewhat limited. Social jetlag, or the frequent shifts in sleep timing due to the discrepancy between sleep schedules on workdays and non-workdays, has been linked to greater BMI and obesity [15, 16]. Other studies have shown that greater night-to-night variability in sleep duration and sleep timing associates with greater BMI and poor food consumption habits that increase risk for weight gain [17–19]. Consistent with these findings, our group previously showed that first-year college students who gained weight reported greater variability in their sleep duration relative to those who did not, a finding independent of average sleep duration [20]. Taken together, these findings argue that regularity of sleep schedules may associate with various markers of obesity risk. However, previous studies have tended to define sleep regularity as how much a person deviates from their average of a given sleep characteristic. Such approaches result in multiple analyses (standard deviation from average sleep duration, wake time, etc.), and by focusing on changes from average, may limit the possibility of capturing more rapid changes in sleep-wake episodes that occur on a day-to-day basis.

In their original paper, Phillips and colleagues presented the sleep regularity index (SRI), an alternate measure of sleep regularity, and validated this measure in a sample of college students [21]. SRI calculates the percentage probability of an individual being asleep (or awake) at the same time point 24 hours apart. Previous sleep regularity metrics, such as interdaily stability and standard deviation measures, assess deviation in sleep patterns from an individual’s average and reflects sleep regularity on a multi-day scale [22]. In contrast, SRI assesses variability in sleep episodes between consecutive days and reflects day-to-day changes in sleep [21, 22]. Undergraduates in the Phillips et al. study with lower SRI scores had a delayed circadian phase, different light exposure patterns, and lower academic performance than those with higher SRI scores [21]. Greater sleep-wake irregularity as indexed by SRI has since been linked to greater risk of obesity status among older adults [23], which brings to question whether this SRI-obesity association may be observable earlier in life among college students.

The current study aims to extend this literature and build upon our previous, subsample report (*N* = 132) on the role of sleep in weight gain among first-year college students with added methods of assessing sleep regularity and circadian alignment [20]. Although our original study examined sleep regularity as defined by deviation scores for several sleep characteristics (total sleep time, bedtime, and wake time), this study focuses on using the sleep regularity index in our complete dataset (*N* = 581). First, we tested whether sleep regularity as indexed by SRI associated with BMI among students at the beginning of the school year and with BMI change (∆BMI) after 9 weeks of their first semester. Second, we aimed to consider the role of circadian phase and circadian alignment as possible contributors to any sleep regularity-obesity association. Thus, in a subset of participants who completed a dim light melatonin onset (DLMO) protocol, we examined whether circadian phase quantified by DLMO and circadian alignment quantified by DLMO-bedtime phase angle associated with BMI and ∆BMI.

## Methods

### Participants

Participants in the current report were drawn from a larger, prospective study that examined sleep among students during their transition into college. The parent study tracked first-year Brown University students and their sleep patterns across the first 9 weeks of the first semester with an on-line daily sleep diary which started the first day of classes and ended before their academic late autumn break. Participants were recruited across 3 years (2012–2014). Each participant’s initial visit included reviewing procedures, providing consent, and obtaining a blood sample for genotyping (data not included in current analyses). Participants provided written consent, and all procedures were approved by the Lifespan Institutional Review Board for Protection of Human Subjects.

### Body mass index (BMI)

Participant height (kg) and weight (m) were measured at the start of classes (T1) and end of 9 weeks (T2). Height was measured with shoes off using a stand-mounted stadiometer; weight was measured without shoes and outer clothing using a calibrated digital scale. BMI was calculated as kg/m^2^. In accordance with CDC guidelines, for participants <20 years old at the start of classes, BMI-for-age percentile was used to determine participants’ weight category at T1. For participants 20 years and older (*n* = 15), as per CDC guidelines, adult criteria were used to determine weight status (underweight, BMI < 18.5, overweight, ≥25 and <30, and obese, ≥30).

### Sleep measures

Participants completed a daily online sleep diary prompted by email. Participants reported their bedtime, wake-time, sleep onset latency, and wake after sleep onset for the previous major sleep episode and noted the occurrence of daytime napping in the previous 24 hours. Missed diary days were coded as missing and not used in the calculation of sleep patterns. Based on the diary reports, total sleep time (TST) was calculated as time spent asleep between bedtime and wake-time. We calculated sleep regularity using the sleep regularity index [21]. Of note, participants were asked to report information about the number and duration, but not timing, of naps and night awakenings. Due to this lack of timing information, SRI was calculated without considering naps or awakenings. The SRI score reflects participants’ sleep regularity across the 9 weeks, where 100 would indicate perfect alignment in the timing of sleep episodes and 0 would indicate completely random timing of sleep episodes.

### Circadian phase and alignment

All participants in the parent study were invited to participate in a circadian phase assessment (DLMO); a subset of participants volunteered to complete this protocol. Full details of the DLMO assessment are described in our previous report [24]. Briefly, 6.5-hour saliva sample collection windows were created surrounding the predicted time of each participant’s DLMO phase, as estimated based on participants’ diary-reported sleep schedules across the preceding 5 nights. Participants came to a large auditorium where light levels were kept below 20 lux. They were seated and permitted to study, talk quietly with friends, or use their laptops (screen brightness was measured and dimmed to <10 lux). Every 30 minutes throughout the collection window, saliva samples were collected using salivettes (Sarstedt, Germany). Saliva samples were centrifuged and chilled immediately then frozen at −20°C within 15 hours. Melatonin concentration in saliva was determined using radioimmunoassay (Alpco) with sensitivity of 0.9 pg/mL, intra-assay coefficient of variation 7.9%, and interassay coefficient of variance 11.7%.

Each participant’s DLMO phase was calculated using a linear interpolation across times bracketing a 4 pg/mL threshold. Circadian alignment was quantified as the phase angle (hours) from their DLMO to average diary-reported bedtime.

### Statistical analyses

Statistical analyses were performed with SPSS 26.0 (IBM Corporation, Armonk NY) for Windows/Apple Mac. Given our own and other previous reports of sex differences [20, 25, 26], we analyzed data with one-way ANOVAs to characterize any sex differences across variables of interest. For descriptive purposes, data were then analyzed with one-way ANOVAs to estimate average T1 weight-group differences in TST, SRI, DLMO, and phase angle. ∆BMI was calculated as the difference between T2 and T1, where a positive value indicates an increase in BMI across the 9 weeks. Data were then analyzed with linear regressions that controlled for sex and average TST to estimate the association between SRI, T1 BMI, and ∆BMI. A second set of analyses estimated the associations between DLMO, bedtime phase angle, BMI, and ∆BMI for those participants in whom DLMO was measured. In all models predicting ∆BMI, T1 BMI was included as a covariate to control for the possible confounding effects that individual differences in baseline BMI may have on change in BMI over time.

## Results

### Participants

A total of 619 freshman college students from the parent study completed sleep diaries (>50% of daily diaries) and were assessed in-person for their height and weight at the start of classes. Of these participants, 605 participants had their height and weight assessed again at the end of nine weeks (T2). Consistent with previous studies [20], we excluded 24 (4.0%) participants from analyses because their recorded height at T2 was shorter (≥1 cm less) than their recorded height at T1, which likely reflects measurement error. A total of 581 participants were included in current analyses that examined TST and SRI. A subset of these participants (*n* = 161) completed the DLMO protocol and were included in analyses that examined DLMO and DLMO-bedtime phase angle.

Of note, presented results are based on calculating SRI without nap or nighttime awakening data, but an alternate SRI calculation was also considered. In order to incorporate available nap and awakening data (i.e., nap duration) without information about the timing of these episodes, a modified SRI calculation was derived to assume zero agreement in the timing of daytime naps on consecutive days. This calculation multiplied sleep and wake index values by the proportion of the sleep or wake period spent in the opposing state. This modified SRI was highly correlated with the unmodified SRI reported here, which omitted nap and daytime data (Pearson *r* = 0.997, p < .001). Results persisted regardless of which SRI calculation was used, and thus only results from the unmodified SRI are presented in the current report.

### Total sleep time, sleep regularity index, and BMI

**Figure 1.**
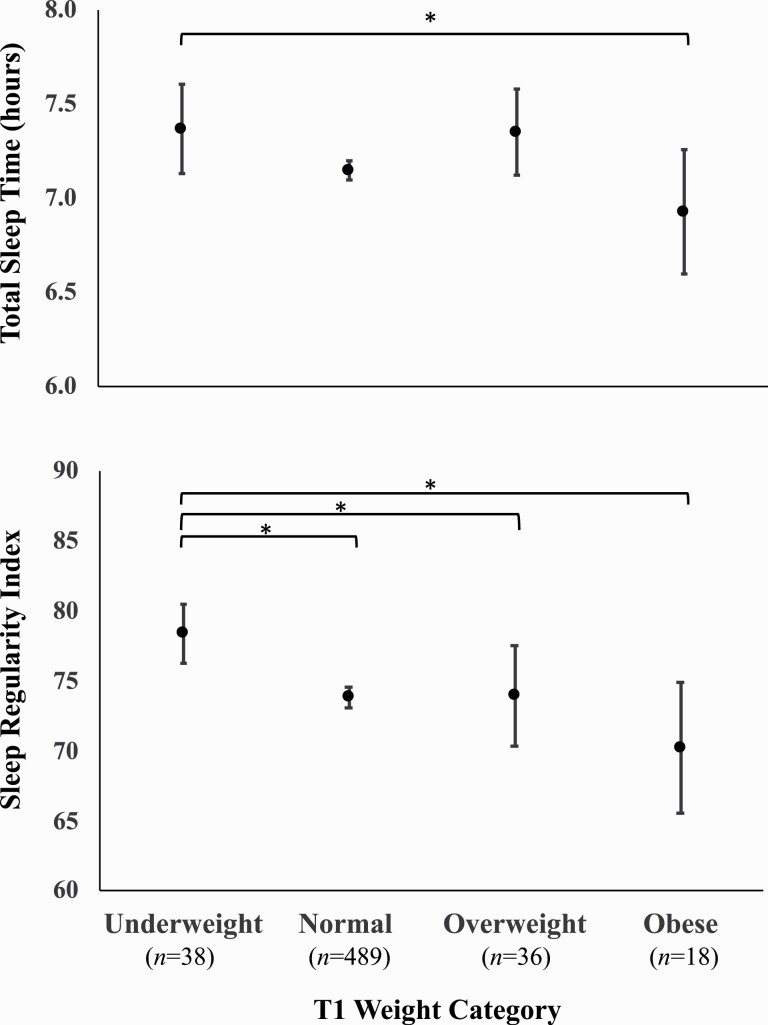
Average total sleep time (TST) and sleep regularity index (SRI) significantly differ by baseline (T1) weight category. Error bars refer to 95% Confidence Intervals. *Significant post-hoc contrast, p < .05.

Table 1 reports participant characteristics and simple correlations with T1 BMI. Simple correlation analyses showed that participant age was not correlated with BMI, average wake time, bedtime, TST, or SRI (p’s > 0.05). Lower SRI was moderately correlated with shorter TST (*r* = 0.24, p < .001). There were several significant sex differences (Table 2). Relative to men, women had an earlier DLMO [*F*(1,159) = 6.15, p = 0.014], slept less [*F*(1,579) = 5.75, p = 0.017] and showed a greater increase in BMI [*F*(1,579) = 4.25, p = 0.040]. As shown in Figure 1, the underweight group showed significantly longer average TST than the obese group [group mean difference = .44 hours (S.E. = 0.19), *t*(577) =2.28, p = 0.023, Figure 1]. The underweight group also had a significantly higher average SRI than each of the other weight groups, with the largest difference being with the obese group [group mean difference = 8.15 (S.E. = 2.46), *t*(577) = 3.31, p = 0.001, Figure 1].

**Table 1.** Participant characteristics

Variable	Mean (SD) or frequency	Minimum	Maximum	*r* correlation with T1 BMI
Age at T1	18.7 ± 0.5 years	18.0	22.0	−.00
Sex	58% Female	—	—	−.01
Race	48% non-white	—	—	
T1 BMI	22.0 (3.5) kg/m^2^	14.9	45.5	—
T2 BMI	22.6 (3.4) kg/m^2^	14.9	44.4	.97**
Change in BMI	0.6 (0.8) kg/m^2^	−2.2	3.9	−.16**
TST	7.2 (0.7) hours	3.6	9.0	−.06
Bed Time	01:36AM (60 minutes)	10:00PM	04:48AM	.05
Wake Time	09:06AM (54 minutes)	03:12AM	11:54AM	.03
Sleep Onset Latency	11.4 (9) minutes	0.0	70.2	−.02
Wake After Sleep Onset	5.4 (6) minutes	0.0	52.2	.06
Daytime Nap	16.8 (17.4) minutes	0.0	120	.10*
DLMO	12:06AM (96 minutes)	07:59PM	04:00AM	.09
Phase Angle	1.7 (1.4)	−4.8	5.7	−.04
Average SRI	74.1 (8.7)	25.7	91.6	−.15**

T1 refers to initial assessment at start of classes; T2 at the end of 9 weeks; BMI, body mass index; TST, total sleep time; DLMO, dim light melatonin onset; Phase Angle, the time between DLMO and bedtime; SRI, sleep regularity index; change in BMI refers to change over 9 weeks, with a positive value indicating an increase in BMI. *p < .05, **p < 0.001

**Table 2.** Sex differences in total sleep time, DLMO, and change in BMI

	Mean (SD)		P-value
	Men	Women	
Age (years)	18.7 (0.5)	18.6 (0.5)	.064
TST (hours)	7.2 (0.7)	7.1 (0.7)	.017
DLMO (clock time)	24.5 (1.5)	23.9 (1.5)	.014
Phase Angle (hour)	1.5 (1.3)	1.8 (1.4)	.160
SRI	73.4 (9.0)	74.5 (8.5)	.114
T1 BMI (kg/m^2^)	22.0 (3.0)	22.0 (3.8)	.866
Change in BMI (kg/m^2^)	0.5 (0.8)	0.6 (0.9)	.04

TST, total sleep time; DLMO, dim light melatonin onset; Phase Angle, the time between DLMO and bedtime; SRI, sleep regularity index; T1 refers to initial assessment at start of classes; BMI, body mass index at T1; change in BMI refers to change over 9 weeks, with a positive value representing an increase in BMI. P-value refers to one-way ANOVA tests for sex differences.

On average, participants gained 1.8 ± 2.4 kg (range: −7.2–11.4). Approximately 6% of participants lost ≥2 kg, 39% gained 2–5kg, and 8% gained >5 kg. Average TST was not correlated with BMI or ∆BMI (*r’s =* −0.07 to −0.06; p’s > 0.05). Greater T1 BMI was correlated with less ∆BMI by T2 (*r* = −0.16, p < .001). Shown in Table 3, lower SRI was associated with greater T1 BMI (*B* = −0.06, p < .001) after controlling for sex and TST (overall model: *R*^2^ = 0.02, p = 0.004). ∆BMI was not significantly associated with SRI (see Table 4; p = 0.062). Exploratory analyses that examined SRI as a categorical predictor, with participants categorized into SRI quartiles, showed the associations between SRI, BMI, and ∆BMI were linear (data not shown).

**Table 3.** Lower sleep regularity index is associated with greater body mass index at T1

	*B* (95% CI)	S.E.	*t*	P-value
Sex	.00 (−.57–.57)	.29	.01	.993
TST	−.11(−.54–.32)	.22	−.50	.621
SRI	−.06 (−.09–−.02)	.02	−3.38	<.001

TST, total sleep time; Sex, male serves as reference group; SRI, sleep regularity index. B refers to the estimated average change in BMI (kg/m^2^) per unit change in each predictor.

**Table 4.** Sleep regularity index is not significantly associated with BMI change overtime

	*B* (95% CI)	S.E.	*t*	P-value
Sex	.12 (−.01–.26)	.07	1.72	.086
TST	−.10 (−.21–.00)	.05	−1.96	.051
T1 BMI	−.04 (−.06–−.02)	−.15	−3.69	<.001
SRI	.01 (.00–.02)	.08	1.87	.062

TST, total sleep time; Sex, male serves as reference group; SRI, sleep regularity index; T1 BMI, body mass index at the beginning of classes; BMI Change (dependent variable), change in BMI after 9 weeks, with a positive value representing an increase in BMI. *B* refers to the estimated average change in the BMI (kg/m^2^) change per unit change in each predictor.

### Circadian phase, circadian alignment, and BMI

On average, later DLMO was correlated with a shorter DLMO-bedtime phase angle (*r* = −0.63, p < .001), shorter TST (*r* = −0.16, p = 0.043), and lower SRI (*r* = −0.35, p < .001). A one-way ANOVA showed that weight groups did not significantly differ in average DLMO or phase angle (p’s > 0.05). Regression analyses showed that there were no significant associations between DLMO or phase angle with T1 BMI or ∆BMI (∆*R*^2^’s = 0.002 to 0.010, p’s > 0.05).

## Discussion

The primary aim of this study was to examine whether sleep-wake regularity as indexed by SRI would associate with BMI and change in BMI over time among first-year college students. In this sample, SRI and TST were modestly correlated. We found that greater sleep-wake irregularity, but not TST, associated with greater BMI at the start of classes. Our finding is consistent with findings from the larger literature that links various forms of sleep irregularity and various forms of circadian disruptions to greater BMI and other indicators of obesity risk.

We found that although greater sleep-wake irregularity was associated with greater BMI, it was not significantly associated with change in BMI over time. Based on the extant literature linking circadian disruptions to weight gain over time, we initially predicted that more sleep irregularity would associate with a greater increase in BMI during the initial transition to college. On average, our participants showed moderate weight gain across the nine weeks. Of note, in this sample, baseline BMI was negatively correlated with BMI change such that having a greater BMI at the start of classes correlated with less of a BMI increase or weight gain after 9 weeks. Most of our participants were normal weight young adults at the start of classes and only 20 (3%) of participants previously normal or underweight became overweight at the end of 9 weeks. None of the non-obese participants became obese in this timeframe. It is possible that in this sample, sleep irregularity was associated with normative growth among these young adults as opposed to unhealthy weight gain. Another interpretation may be that the 9-week interval between our BMI assessments was short relative to other longitudinal studies examining obesity risk, and future studies are warranted to examine how sleep regularity may associate with longer term changes in weight.

Sleep-wake irregularity may associate with BMI partly due to underlying circadian disruptions. Previous findings showed college students with lower SRI had a delayed circadian phase as quantified by DLMO and irregular light exposure patterns [21], suggesting that sleep irregularity co-occurred with delayed circadian rhythms and could perpetuate circadian misalignment. In the current study, we similarly observed a correlation between greater irregularity (lower SRI) and later DLMO. However, we did not find significant associations between DLMO or DLMO-bedtime phase angle and BMI. Out of 161 participants who completed the DLMO protocol, only 13 were overweight and 3 were obese. Our nonsignificant findings may partly reflect the lack of variability in BMI in the available participant subset.

Regarding sex differences, we found that women slept less and had an earlier DLMO than men. Although not statistically significantly, women showed a greater DLMO-sleep onset interval than men. These observed sex differences are consistent with previous reports [27–29]. Additionally, we found that there were no baseline sex differences in BMI but women in our sample showed slightly greater weight gain and increase in BMI. In our initial report by Roane and colleagues [20], we found a significant sex difference such that variability in sleep duration and bedtime associated with greater weight change specifically in men but not in women [20]. Although we did not test for possible moderating effects of sex due to limited statistical power in the current study, future studies are needed to further consider these sex differences and whether sex moderates the associations between sleep regularity, DLMO, and BMI.

Although we did not find a significant main effect of sleep regularity on change in BMI, it is possible that individual characteristics moderate this relationship. In addition to aforementioned sex differences, future studies are needed to consider how participant demographic, sleep, and circadian characteristics may moderate any effects of sleep regularity on BMI and change in BMI over time. For instance, obesity risk is known to differ as a function of race, chronotype, circadian phase, and sleep duration [30–35]. Future studies are needed to consider whether these factors may affect individual vulnerability to any effects of sleep irregularity on obesity risk.

Strengths of the current study include the combined collection of daily sleep diaries across nine weeks, which allowed for deep phenotyping of sleep patterns, along with prospective assessment of BMI in a large sample of college students. In addition, a subset of the sample completed a DLMO assessment which allowed for further study of circadian phase and alignment with bedtime in relation to BMI.

The current study has several limitations. First, because there was limited variability in weight and BMI among this subset, we may have been underpowered to detect these latter associations. Although our sleep diary collection was thorough, the study collected information on the duration of naps and nighttime awakenings but not the timing of these episodes. The presented SRI and results in this report omitted nap and nighttime awakenings, though our modified calculation (see Results) was highly correlated and results persisted regardless of which approach was used. Even so, it is possible that our estimates of SRI do not fully reflect the degree of sleep irregularity in the sample. In addition, subjective reports of sleep are subject to recall bias and correlations between participant reports and actigraphy vary based on sleep characteristics. For instance, studies have found participants tend to overestimate their sleep duration, whereas their reports of sleep timing are moderately to highly correlated with actigraphy [36–40]. The current study involved a thorough, 9-week daily assessment period which likely reduced confounds of participant recall bias, but the subjective nature of this method remains a possible limitation. Finally, we examined students’ sleep during their first college semester to focus on their initial transition to college, but it is possible that differences in their college sleep patterns relative to their sleep before matriculation may have meaningful impact on weight gain that was not captured in the current study. Future studies are warranted to consider these factors and examine how sleep regularity may relate to longer-term changes in BMI throughout young adulthood.
